# Alteration of Angiopoietin-Like Protein 4 Levels in Serum or Urine Correlate with Different Biochemical Markers in Hyperlipidemia-Related Proteinuria

**DOI:** 10.1155/2020/5281251

**Published:** 2020-03-23

**Authors:** Xia Gao, Min Zhang, Weidong Feng, Zichuan Xu, Yan Wang, Lingna Shi, Jing Zhang

**Affiliations:** ^1^Nephrology Department, Guangzhou Women and Children's Medical Center, Guangzhou City 510623, China; ^2^Graduate School, Ningxia Medical University, Yinchuan City 750000, China

## Abstract

Angiopoietin-like protein 4 (ANGPTL4) is widely known as a key regulator of lipid metabolism. We investigated the relationship between ANGPTL4 expression in serum or urine and blood lipid or urine protein levels of patients with hyperlipidemia- (HL-) related proteinuria. Sixty-eight patients with HL-related proteinuria (HL-Pro group), 68 patients with HL without proteinuria (HL-NPro group), 46 patients with non-HL-related proteinuria (NHL-Pro group), and 50 healthy control (Con) subjects were selected. There were no significant differences in serum ANGPTL4 levels between the Con group (36.82 ± 17.03 ng/ml) and the HL-Pro group (27.94 (18.90, 53.72) ng/ml). Additionally, the serum ANGPTL4 levels in the HL-Pro group were significantly lower than those in the HL-NPro group (53.32 ± 24.01 ng/ml) (*P* < 0.001). The urine ANGPTL4/Cr levels in the HL-Pro group (52.01 (45.25, 79.79) *μ*g/g) were significantly higher than those in the HL-NPro group (9.96 (8.35, 12.43) ng/ml) (*P* < 0.05). A significant alteration in urine ANGPTL4/Cr levels was observed in the NHL-Pro group (69.41 ± 55.36 *μ*g/g) and the Con group (10.08 ± 2.38 *μ*g/g) as well. There was no correlation between serum and urine ANGPTL4 levels of the four groups (*P* > 0.05). Serum ANGPTL4 levels (HL-Pro/HL-NPro group) were positively correlated with total cholesterol (TC) and triglyceride (TG) levels in hyperlipidemia patients. However, there was no correlation between urinary ANGPTL4 levels and TC or TG (*P* > 0.05). Urine ANGPTL4 levels were positively correlated with 24hUPro in patients with renal impairment (HL-Pro/NHL-Pro group). To summarize, ANGPTL4 may be considered an accurate predictor of proteinuria with HL. Notably, serum or urine ANGPTL4 levels indicated the degree of proteinuria or hyperlipidemia, respectively, in HL patients.

## 1. Introduction

It was first confirmed more than 30 years back that the apolipoprotein APOA1 and APOB had a role in renal expression [1]. In 1982, Moorhead et al. first proposed the hypothesis of “lipid nephrotoxicity,” suggesting that hyperlipidemia (HL) could cause kidney disease and participate in its incidence and development [2]. Ineffective lipid accumulation and total cholesterol (TC) transport in kidney accelerates the incidence of glomerular sclerosis and renal interstitial fibrosis and is one of the important mechanisms underlying kidney damage and subsequent proteinuria [3]. Recent studies have demonstrated that glomerulus mesangial cells and podocytes could express lipid metabolism proteins, suggesting that the kidney is one of the sources of lipoprotein secretion as well [4].

Angiopoietin-like protein 4 (ANGPTL4) was first discovered in 2000 [5] as a novel member of the angiopoietin family. Under physiological conditions, ANGPTL4 functions as a key regulator of lipid metabolism and its role has been widely reported in metabolic diseases and cardiovascular diseases [6, 7]. In addition, some studies have reported that ANGPTL4 plays a significant role in tumor invasion, intestinal microecological regulation, fracture healing, and tendon cell proliferation, adhesion, and migration [8–11]. ANGPTL4 involvement in kidney disease was first studied in 2011. Clement et al. [12–14] confirmed that sialylated ANGPTL4 is secreted into the blood by peripheral organs, subsequently inhibiting LPL activity and inducing hypertriglyceridemia. Meanwhile, sialylation-deficient ANGPTL4 is secreted by podocytes and is involved in proteinuria development in the nephrotic animal model.

We aimed to investigate whether ANGPTL4 is a reliable predictor of the degree of proteinuria in HL patients. We first analyzed the possible correlation between serum or urine ANGPTL4 levels and the primary indicators of serum lipids in patients with HL-related proteinuria; we further explored the possible relationship between serum or urine ANGPTL4 and urinary protein levels. This study serves as a source of new clinical data and molecular targets for future research on HL-related proteinuria.

## 2. Methods

### 2.1. Research Selection Criteria

#### 2.1.1. Inclusion Criteria for HL

(1) TC ≥ 6.2 mmol/l (240 mg/d1); (2) triglyceride (TG) ≥ 2.3 mmol/l (200 mg/d1); (3) low density lipoprotein cholesterol (LDL‐C‐C) ≥ 4.1 mmol/L (160 mg/d1); (4) the above 3 markers should be present at comparable levels, consistent with the 2016 Chinese adult dyslipidemia prevention guideline (revised edition 2016) diagnostic classification standard [15].

#### 2.1.2. Inclusion Criteria for Proteinuria

The standards for inclusion of proteinuria patients in this study are as follows: 24 h urine protein (24hUPro) > 150 mg or 24‐hour urinary albumin > 30 mg; no other renal impairment in patients. 24hUPro quantitative grading standard [16]: grade 0: 0~0.15 g, grade 1: 0.15~0.5 g, grade 2: 0.5~1.0 g, grade 3: 1.0~3.5 g, and grade 4: >3.5 g.

#### 2.1.3. Exclusion Criteria

(1) Patients for whom detailed inquiry of medical history, physical examination, and necessary laboratory tests revealed diagnosis of nephrotic syndrome, diabetes, hypothyroidism, liver disease, renal failure, systemic lupus erythematosus, glycogen accumulation, myeloma, fat atrophy, acute porphyria, and above average use of certain drugs, such as glucocorticoids, diuretics, and other drugs that disturb the metabolism of blood lipids; (2) patients with tumors in various organ systems; and (3) patients or respective guardians who failed to provide informed consent were excluded.

### 2.2. Patients

#### 2.2.1. Case Group

One hundred and eighty-two patients hospitalized in Gansu Provincial People's Hospital between January 2017 and December 2018 were enrolled in the group: 68 patients (36 males and 32 females) in the HL-Pro group, 68 patients (48 males and 20 females) in the HL-NPro group, and 46 patients (36 males and 10 females) in the NHL-Pro group who met the inclusion and exclusion criteria for hospitalization were selected randomly as subjects in this study.

#### 2.2.2. Control Group

In addition, 50 healthy individuals from the Physical Examination Center of Gansu Provincial People's Hospital were selected for the Con group, including 30 males and 20 females and excluding diseased individuals. Members of the healthy control group had TC < 6.2 mmol/l and TG < 2.3 mmol/l.

### 2.3. Serum and Urine Collection

Blood and urine samples from patients and controls were centrifuged for 5 min at 2400 rpm. Serum and urine samples were collected and stored at -80°C until analysis.

### 2.4. Serum and Urine ANGPTL4

ANGPTL4 concentration in serum and urine samples was measured using the ELISA kit of human ANGPTL4 (Jiangsu Sofia Biotechnology Co., LTD., batch number: MM-1759H1) following the manufacturer's instructions.

### 2.5. Clinical Biochemical Indices

TC, TG, LDL-C, high-density lipoprotein (HDL-C), Blood Urea Nitrogen (BUN), Serum Creatinine (SCr), and 24hUPro levels were determined using the automatic biochemical analyzer of the laboratory at Gansu Provincial People's Hospital.

### 2.6. Statistical Methods

The experimental data were subjected to statistical processing using the SPSS 20.0 software. Qualitative data (gender) were expressed in terms of frequency and percentage (*n*, %), and comparisons between groups were performed using *χ*^2^ tests. The Kolmogorov-Smirnov method was used for the normality testing of the quantitative data. The quantitative data, with continuity consistent with the normal distribution, were expressed in terms of mean and standard deviation (−*x* ± *s*). Quantitative data for nonnormal distributions were expressed in terms of median and interquartile range *P*50 (*P*25, *P*75).

The Levene method was used to test the homogeneity of variance. The quantitative data with normal distribution and variance were compared using two independent sample *t* tests. Comparative analysis between the groups was conducted using one-way ANOVA. Quantitative data of nonnormal distribution or variance between the two groups were compared using the Mann-Whitney *U* rank sum test, and the comparison between groups was performed using the Kruskal-Wallis test. Spearman correlation analysis was used to determine the correlation between the indicators. The test level used was *α* = 0.05.

## 3. Results

### 3.1. Comparison of the Basic Characteristics of the Four Subject Groups

There was no difference in systolic and diastolic pressure and gender distribution between the four groups (*P* > 0.05). There was no difference in age between the HL-Pro, HL-NPro, and NHL-Pro groups (*P* > 0.05). The average age of the Con group was lower than that of the other three groups, and the difference was statistically significant (*P* < 0.05), suggesting that the incidence of dyslipidemia and proteinuria increased with age in a statistically significant manner (*P* < 0.05). The TC and TG levels of the HL-Pro and HL-NPro groups were significantly higher than those of the Con and NHL-Pro groups (*P* < 0.05). The renal damage indices associated with SCr and 24hUPro were significantly higher in the HL-Pro and NHL-Pro groups than those in the other two groups (*P* < 0.05). See Table 1.

### 3.2. Quantitative Detection Results and Correlation Analysis of Serum and Urine ANGPTL4 Levels of the Four Groups

The serum ANGPTL4 levels in the HL-Pro group (27.94 (18.90, 53.72) ng/ml) did not differ significantly from those in the Con group (36.82 ± 17.03 ng/ml) (*P* > 0.05); however, the serum ANGPTL4 levels of the HL-NPro group (53.32 ± 24.01 ng/ml) increased significantly (*P* < 0.05), while there was a significant decrease of the same in the NHL-Pro group (23.56 ± 9.30 ng/ml) (*P* < 0.05). Further analysis of both HL groups revealed that the serum ANGPTL4 levels in the HL-Pro group were significantly lower than those in the HL-NPro group (*P* < 0.05). Additionally, the HL-Pro group had higher serum ANGPTL4 levels than the NHL-Pro group (*P* < 0.05). See Tables 2 and 3.

Analysis of the characteristics of urine ANGPTL4 levels in different groups revealed the following: compared to the urine ANGPTL4 levels in the Con group (10.08 ± 2.38 ng/ml), a significant elevation was observed in the HL-Pro group (52.01 (45.25, 79.79) ng/ml) and the NHL-Pro group (69.41 ± 55.36 ng/ml), while a significant reduction was observed in the HL-NPro group (9.96 (8.35, 12.43) ng/ml) (*P* < 0.05). Further analysis revealed that the urine ANGPTL4 levels in the HL-Pro group were significantly higher than those in the HL-NPro group (*P* < 0.05). There was no difference in the urine ANGPTL4 levels between the HL-Pro group and the NHL-Pro group (*P* > 0.05). The specific values are presented in Tables 2 and 3. Lastly, the correlation analysis of serum and urine ANGPTL4 expression levels of the four groups revealed no correlation (*P* > 0.05). See Table 2.

### 3.3. Correlation Analysis of the Serum ANGPTL4 Expression Levels and the Major Clinical Indicators in the Subject Groups

This study investigated the relationship between serum ANGPTL4 and BMI, TC, TG, HDL-C, and LDL-C in the four subject groups. The results are presented in Table 4: in HL patients with or without proteinuria (HL-Pro/HL-NPro), serum ANGPTL4 and TC (*r* = 0.483, *P* = 0.001; *r* = 0.445, *P* < 0.001) and TG (*r* = 0.351, *P* = 0.002; *r* = 0.583, *P* < 0.001) levels were positively correlated. Among non-HL patients (NHL-Pro/Con), serum ANGPTL4 levels were positively correlated with body mass index (BMI) (*r* = 0.466, *P* = 0.001) in the NHL-Pro group and with ANGPTL4 and BUN, SCr, and 24hUPro in the NHL-Pro/Con group. There was no correlation between glomerular filtration ratio (GFR) and other indicators (*P* > 0.05). See Table 4.

### 3.4. Serum and Urine ANGPTL4 Expression in Patients with HL (HL-Pro/HL-NPro Group) among the Different TC Classification Groups

The TC levels of the HL-Pro and HL-NPro groups were graded by a quartile spacing method, as shown in Table 5. There was a difference in serum ANGPTL4 expression at different TC levels in the HL-Pro group, while there was no increase in serum ANGPTL4 expression with an increase in TC levels. However, the serum ANGPTL4 levels in the HL-NPro group increased simultaneously with an increase in TC, and the difference between the graded groups was statistically significant (*P* < 0.05). The difference in urine ANGPTL4 levels and TC levels in the HL-Pro group was statistically significant (*P* < 0.05). There was no significant difference in urine ANGPTL4 levels at different TC levels in the HL-NPro group (*P* > 0.05).

### 3.5. Serum and Urine ANGPTL4 Expression in Patients with HL (HL-Pro/HL-NPro Group) Based on Different TG Classification Groups

The TG levels of the HL-Pro group and the HL-NPro group were graded by the quartile method, as shown in Table 6. The HL-Pro and HL-NPro groups exhibited an increase in serum ANGPTL4 expression with an increase in TG, and there was a statistically significant difference between the graded groups (*P* < 0.05). However, there was no significant difference in urine ANGPTL4 levels between the two groups (*P* > 0.05).

### 3.6. Correlation Analysis of Urine ANGPTL4 Expression Levels and the Major Clinical Indicators in the Subject Groups

Considering that there is no correlation between urine and serum ANGPTL4 levels, this study further analyzed the relationship between urinary ANGPTL4 levels and its major clinical indicators. As indicated in Table 7, there was no correlation between urine ANGPTL4 and BMI, TC, TG, HDL-C, and LDL-C among the four groups (*P* > 0.05), suggesting that there was no correlation between urine ANGPTL4 expression and serum lipid levels.

Further analysis of the correlation between urine ANGPTL4 and renal damage indicators revealed positive correlation between the levels of urine ANGPTL4 and BUN (*r* = 0.273, *P* = 0.012; *r* = 0.385, *P* = 0.004), SCr (*r* = 0.339, *P* = 0.002; *r* = 0.463, *P* = 0.001), and 24hUPro (*r* = 0.388, *P* = 0.001; *r* = 0.451, *P* = 0.001) between the two groups (HL-Pro/NHL-Pro) with renal impairment. However, the levels of urine ANGPTL4 and GFR (*r* = −0.263, *P* = 0.015; *r* = 0.550, *P* < 0.001) between the two groups (HL-Pro/NHL-Pro) had negative correlation. These results suggest that urine ANGPTL4 expression is associated with increased impairment of the patient's major renal function indicators (BUN, SCr, 24hUPro, and GFR).

### 3.7. Serum and Urine ANGPTL4 Expression in Proteinuria Patients (HL-Pro/NHL-Pro Group) among the Different 24hUPro Classification Groups

We divided the 24hUPro into 5 grades, as previously mentioned: 0-0.15 g, 0.15-0.5 g, 0.5-1.0 g, 1.0-3.5 g, and >3.5 g, for the two groups of proteinuria patients (HL-Pro and NHL-Pro) [8]. With the increase of 24hUPro in the HL-Pro and NHL-Pro groups, the urine ANGPTL4/Cr expression followed an increasing trend, with statistically significant differences between the graded groups (*P* < 0.05). However, there was no significant difference in serum ANGPTL4 expression between the two groups at different 24hUPro levels (*P* > 0.05). See Table 8.

### 3.8. Serum and Urine ANGPTL4 Expression in Proteinuria Patients (HL-Pro/NHL-Pro Group) from Different GFR Classification Groups

The GFR levels in the HL-Pro and NHL-Pro groups were divided into the following 5 groups: normal phase 1 or elevation ≥ 90 ml/min·1.73m^2^, phase 2 mild 60-89 ml/min·1.73m^2^, phase 3 moderate 30-59 ml/min·1.73m^2^, phase 4 severe 15-29 ml/min·1.73m^2^, and phase 5 < 15 ml/min·1.73m^2^. As indicated by the data in Table 9, there was a significant difference in serum ANGPTL4 expression between the HL-Pro patients at different GFR levels (*P* < 0.05), while there was no difference in urine ANGPTL4/Cr levels between the groups (*P* > 0.05). Compared to the HL-Pro group, there was no significant difference in serum ANGPTL4 levels between the graded groups in the NHL-Pro group (*P* > 0.05), and the differences in urine ANGPTL4/creatinine levels were statistically significant (*P* < 0.05).

## 4. Discussions

It is widely known that abnormal lipid metabolism mediates glomerular injury, as well as tubulointerstitial glomerular basement membrane thickening and renal interstitial fibrosis, ultimately resulting in the incidence of uremia [17].

However, the mechanism underlying the induction of proteinuria by lipid molecules remains unelucidated. As a key molecule involved in lipid regulation, ANGPTL4 plays a part in the incidence of proteinuria in HL patients, which is the basis of this study as well. We compared the serum and urine ANGPTL4 expression in HL-related proteinuria, as well as in the non-HL-related proteinuria and normal control groups. In addition, we analyzed the possible correlation between ANGPTL4 levels and the major indices of renal damage and blood lipid levels.

A previous study [18] has confirmed that mice with high ANGPTL4 expression have significantly increased levels of TG as well; this was accompanied by hepatomegaly, marginally reduced blood glucose levels, and an increased glycogen content in the liver. In ANGPTL4-knockout mice, a significant decrease in TG and VLDL-C-C levels was associated with increased LPL activity [19, 20]. The study by Zhu et al. [21] suggested that the acquisition and loss of ANGPTL4 function in the mouse model lead to an increase and decrease in TG levels, respectively. In recent years, several studies have confirmed that ANGPTL4 is closely related to lipid metabolism in humans. ANGPTL4 can delay the clearance of TG in the circulation by inhibiting LPL, leading to hypertriglyceridemia [22]. A study of 1,770 diabetic Caucasian individuals displaying metabolic syndrome [21] revealed that serum ANGPTL4 levels were positively correlated with TG levels, while there was a significantly negative correlation with LDL-C and HDL-C.

The present study focused on the relationship between serum lipid levels and serum ANGPTL4 in patients with HL (HL-Pro/HL-NPro) as well. Notably, the results revealed that while TC and TG levels were significantly increased in the HL-Pro group compared to the Con group, there was no significant difference in ANGPTL4 expression levels between the two groups. This might be attributed to the molecular weight of ANGPTL4 (approximately 45-65 kDa) in circulation, which is considerably low for a protein. It is likely that with the aggravation of proteinuria in HL-Pro patients, ANGPTL4 in the circulation leaks out through the glomerular filtration membrane, leading to comparable ANGPTL4 levels between the HL-Pro and Con groups. However, in patients with HL without proteinuria (HL-NPro group), the serum ANGPTL4 level was significantly higher than that in the Con groups, and a significant increase in the blood lipid levels was observed concurrently. Our results confirmed the role of ANGPTL4 in HL incidence as well.

Furthermore, the serum ANGPTL4 levels in the HL-NPro group increased simultaneously with the increase in TC and TG levels. There was a statistically significant difference between the grading groups (*P* < 0.05); however, this trend was not observed in the HL-Pro group. These data indicate that ANGPTL4 is a sensitive indicator of blood lipid level alterations in HL patients. However, in patients with renal impairment due to glomerular filtration barrier dysfunction, serum ANGPTL4 levels decreased significantly (HL-Pro vs. HL-NPro), suggesting that serum ANGPTL4 is not an ideal indicator for assessing abnormal lipid metabolism in HL-Pro patients.

In 2015, Ma et al. [23] revealed for the first time that high ANGPTL4 expression in the glomerular tissue was detectable in urine, and the ANGPTL4 in urine was secreted by podocytes, regardless of ANGPTL4 levels in the blood. The unique form of ANGPTL4 secreted by podocytes is incapable of entering the blood circulation. This study further confirms the observations made by Ma et al. in rats. On further analysis of the four groups, we observed no correlation between HL-Pro patients with respect to urinary ANGPTL4 and TC or TG levels, which is consistent with Clement's conclusion in a minimally pathological rat model. The lack of correlation between serum ANGPTL4 and urine ANGPTL4 levels may be attributed to the different sources of serum and urine ANGPTL4. Serum ANGPTL4 is primarily secreted by peripheral skeletal muscles and adipose tissues among others, while urine ANGPTL4 is secreted by glomerular podocytes.

Analysis of the relationship between urine ANGPTL4 and the major indicators of blood lipids in the four groups revealed no correlation. This could possibly be attributed to the fact that urine ANGPTL4 does not enter the blood circulation after its secretion by podocytes.

Recent studies have revealed that ANGPTL4 is a ligand for peroxisome proliferator-activated receptors (PPAR*γ* and PPAR*α*). PPAR*γ* agonists inhibit glomerular sclerosis by reducing ANGPTL4 levels [24], while PPAR*α* reduces ANGPTL4 levels and relieves tubulointerstitial damage [25]. Urinary ANGPTL4 levels can indirectly indicate the degree of damage to glomerular podocytes. This study revealed that an increase in 24hUPro expression corresponded to the increase in the expression levels of urine ANGPTL4/Cr, suggesting that ANGPTL4 secreted by podocytes may be involved in the development of HL-related proteinuria. Additionally, we observed that the urine ANGPTL4 levels in the two groups of patients with proteinuria (HL-Pro/NHL-Pro) were significantly correlated with the degree of increase in BUN, SCr, and GFR levels. Our results suggest that the renal damage induced by ANGPTL4 may not just result in the damage to podocytes; they might affect the function of other glomerular solid cells and renal tubules and may be involved in the incidence of renal interstitial inflammation. We are currently performing studies on gene knockout and transgenic animal model of ANGPTL4, including high-throughput sequencing analysis, to provide data for further investigation of the molecular mechanism underlying the role of ANGPTL4 in HL-related proteinuria.

Our study confirmed that there is no correlation between serum and urine ANGPTL4 expression in HL-related proteinuria. Serum ANGPTL4 was significantly associated with blood lipid levels, while it was not associated with renal damage-related indicators. Urine ANGPTL4 is associated with the advancement of renal damage, especially proteinuria, and had no associated changes in lipid levels. Our findings suggest that serum and urine ANGPTL4 should be treated as independent entities in future research.

## 5. Conclusions

There is a significantly positive correlation between serum ANGPTL4 and blood lipid levels; however, there is no association of the same with 24hUPro. Urinary ANGPTL4 levels are positively correlated with the degree of renal damage and have no effect on blood lipid levels. ANGPTL4 might play a significant role in the mechanism underlying proteinuria in hyperlipidemia patients.

## Figures and Tables

**Table 1:** Comparison of the basic conditions of the four groups of subjects.

Group	HL-Pro group (*n* = 68)	HL-NPro group (*n* = 68)	NHL-Pro group (*n* = 46)	Con group (*n* = 50)	Statistics	*P*
Male (*n*, %)	36 (52.94)	48 (70.59)	26 (56.52)	30 (60.00)	4.823	0.185
Age (age)	57.29 ± 14.38	56.44 ± 14.52	57.17 ± 17.58	39.44 ± 16.56	36.398	<0.001
Systolic pressure (mmHg)	137.00 ± 1.93	132.91 ± 2.34	135.43 ± 2.53	128.87 ± 2.19	6.889	0.076
Diastolic pressure (mmHg)	85.74 ± 1.84	90.00 (77.00, 90.00)	80.00 (70.00, 100.00)	79.96 ± 1.60	6.457	0.091
BMI (kg/m^2^)	25.97 ± 3.85	26.33 ± 3.05	25.67 ± 4.15	21.29 ± 3.01	70.299	<0.001
TC (mmol/l)	5.63 ± 1.78	5.36 ± 1.38	4.24 ± 0.17	4.89 ± 0.73	45.428	<0.001
TG (mmol/l)	3.48 (2.39, 7.21)	3.55 (2.68, 4.96)	1.23 ± 0.39	1.11 (0.85, 1.39)	156.385	<0.001
HDL (mmol/l)	0.98 (0.86, 1.12)	1.03 (0.87, 1.25)	0.90 ± 0.26	1.14 ± 0.29	23.567	<0.001
LDL (mmol/l)	2.41 (1.84,3.17)	3.11 ± 1.16	2.17 ± 0.64	1.90 ± 0.37	57.933	<0.001
BUN (mmol/l)	6.25 (5.20, 7.70)	5.60 (4.30, 7.70)	5.50 (4.70, 11.40)	5.48 ± 1.62	7.740	0.052
SCr (*μ*mol/l)	77.56 ± 29.73	70.15 (62.10, 77.10)	77.90 (63.60, 130.10)	63.71 ± 12.11	21.563	<0.001
24hUPro (g/d)	0.34 (0.15, 1.18)	0.08 ± 0.06	0.52 (0.24, 1.17)	0.02 (0.01, 0.09)	152.393	<0.001
GFR (ml/min·11.73 m^2^)	105.84 ± 44.19	108.71 ± 38.79	87.81 ± 41.07	116.92 ± 20.78	13.423	0.004

**Table 2:** Quantitative test results and correlation analysis of ANGPTL4 in serum and urine in four groups of subjects.

Group	Serum ANGPTL4 (ng/ml)	Urine ANGPTL4/Cr (*μ*g/g)	*r*	*P*
HL-Pro group	27.94 (18.90, 53.72)	52.01 (45.25, 79.79)	-0.158	0.099
HL-NPro group	53.32 ± 24.01	9.96 (8.35, 12.43)	0.176	0.076
NHL-Pro group	23.56 ± 9.30	69.41 ± 55.36	-0.149	0.161
Con group	36.82 ± 17.03	10.08 ± 2.38	0.200	0.082

**Table 3:** Comparison of serum and urine ANGPTL4 in two groups of subjects.

Project	Serum ANGPTL4		Urine ANGPTL4/Cr	
	*t*/*z*	*P*	*t*/*z*	*P*
HL-Pro group vs. HL-NPro group	0.000	<0.001^b^	3200.000	<0.001^b^
HL-Pro group vs. NHL-Pro group	1180.000	0.027^b^	1448.000	0.503^b^
HL-Pro group vs. Con group	1836.000	0.459^b^	0.000	<0.001^b^
HL-NPro group vs. Con group	17.225	<0.001^a^	1598.000	0.579^b^
NHL-Pro group vs. Con group	21.848	<0.001^a^	57.377	<0.001^a^

Note: a: independent sample *t* test; b: Mann-Whitney *U* test.

**Table 4:** Correlation analysis between the expression levels of serum ANGPTL4 and the observed indicators in the four groups.

Factor	HL-Pro group		HL-NPro group		NHL-Pro group		Con group	
	*r*	*P*	*r*	*P*	*r*	*P*	*r*	*P*
BMI	0.079	0.262	-0.002	0.494	0.466	0.001	-0.130	0.185
TC	0.483	<0.001	0.445	<0.001	0.081	0.296	0.160	0.133
TG	0.351	0.002	0.583	<0.001	0.177	0.120	0.041	0.389
HDL	-0.048	0.348	0.195	0.057	0.017	0.455	-0.023	0.473
LDL	0.120	0.164	0.530	<0.001	0.050	0.370	0.169	0.120
BUN	0.106	0.195	-0.020	0.435	-0.142	0.173	0.089	0.269
SCr	0.001	0.496	0.124	0.156	-0.220	0.071	0.124	0.195
24hUPro	0.121	0.162	0.158	0.099	-0.076	0.380	-0.160	0.133
GFR	-0.081	0.257	-0.030	0.405	0.153	0.155	0.093	0.260

**Table 5:** Expression of ANGPTL4 in serum and urine in patients with HL (HL-Pro/HLN-Pro group) among the different TC classification groups.

Group (*n*)	TC (*n*) (mmol/l)	Serum ANGPTL4 (ng/ml)	Urine ANGPTL4/Cr (*μ*g/g)
HL-Pro group (*n* = 68)	<4.48 (16)	19.58 (17.99, 22.16)	53.97 (49.07, 62.08)
	4.48-5.81 (20)	24.25 (16.33, 34.51)	75.11 (52.05, 83.85)
	5.81-6.77 (16)	71.44 (45.65, 94.63)	48.53 (40.51, 62.01)
	>6.77 (16)	35.42 (20.00, 69.47)	45.46 (42.51, 96.09)
	*c*2	26.160	10.697
	*P*	<0.001	0.013

HL-NPro group (*n* = 68)	<4.29 (16)	40.40 ± 19.26	8.67 (8.04, 10.28)
	4.29-5.40 (18)	46.56 ± 16.60	10.16 (9.67, 10.99)
	5.40-6.64 (18)	56.76 ± 22.99	10.18 (9.34, 11.48)
	>6.64 (16)	69.97 ± 27.37	12.46 (7.94, 22.26)
	*F*/*c*2	5.699	3.892
	*P*	0.002	0.273

**Table 6:** Expression of ANGPTL4 in serum and urine in patients with HL (HL-Pro/HLN-Pro group) among the different TG classification groups.

Group (*n*)	TG (*n*) (mmol/L)	Serum ANGPTL4 (ng/ml)	Urine ANGPTL4/Cr (*μ*g/g)
HL-Pro group (*n* = 68)	<2.39 (16)	18.76 (17.31, 33.42)	47.10 (45.25, 57.73)
	2.39-3.48 (18)	24.25 (20.09, 41.11)	51.00 (45.79, 76.34)
	3.48-7.21 (18)	21.31 (18.82, 49.67)	62.94 (52.64, 94.32)
	>7.21 (16)	52.03 (34.48, 98.07)	51.89 (36.34, 79.45)
	*c*2	12.819	6.191
	*P*	0.005	0.103

HL-NPro group (*n* = 68)	<2.68 (16)	38.34 ± 18.65	10.42 (9.34, 10.68)
	2.68-3.55 (18)	43.46 ± 21.84	8.99 (8.06, 9.91)
	3.55-4.96 (18)	56.97 ± 15.55	13.76 (8.82, 23.09)
	>4.96 (16)	75.28 ± 22.91	10.11 (7.14, 13.43)
	*F*/*c*2	11.202	7.787
	*P*	<0.001	0.050

**Table 7:** Correlation analysis between the expression levels of urinary ANGPTL4 and the observed indicators in the four groups.

Factor	HL-Pro group		HL-NPro group		NHL-Pro group		Con group	
	*r*	*P*	*r*	*P*	*r*	*P*	*r*	*P*
BMI	-0.164	0.091	-0.071	0.282	-0.369	0.006	0.130	0.184
TC	-0.168	0.085	0.193	0.057	0.022	0.442	-0.125	0.193
TG	0.086	0.243	0.117	0.171	0.084	0.289	-0.077	0.297
HDL	-0.057	0.322	0.078	0.263	-0.220	0.071	0.087	0.096
LDL	0.012	0.463	0.168	0.086	-0.707	<0.001	-0.016	0.456
BUN	0.273	0.012	-0.073	0.276	0.385	0.004	-0.022	0.439
SCr	0.339	0.002	-0.050	0.344	0.463	0.001	-0.201	0.081
24hUPro	0.388	0.001	0.200	0.051	0.451	0.001	0.025	0.433
GFR	-0.263	0.015	0.053	0.335	-0.550	<0.001	-0.081	0.288

**Table 8:** Expression of ANGPTL4 in serum and urine in patients with proteinuria (HL-Pro/NHL-Pro group) among the different 24hUPro classification groups.

Group (*n*)	24hUPro (*n*) (g/d)	Serum ANGPTL4 (ng/ml)	Urine ANGPTL4/Cr (*μ*g/g)
HL-Pro group (*n* = 68)	Level 0 (16)	56.70 (23.16, 98.07)	48.99 (44.22, 66.65)
	Level 1 (28)	18.75 (17.15, 53.72)	47.20 (45.25, 56.32)
	Level 2 (4)	26.05 (20.27, 31.83)	51.31 (51.00, 51.62)
	Level 3 (16)	35.42 (22.14, 46.83)	89.41 (52.72, 112.99)
	Level 4 (4)	29.84 (22.76, 36.91)	84.43 (80.02, 88.84)
	*c*2	14.726	18.461
	*P*	0.005	0.001

NHL-Pro group (*n* = 46)	Level 1 (22)	24.33 (18.10, 33.79)	40.18 (27.98, 64.18)
	Level 2 (8)	19.75 (18.78, 24.25)	70.11 (32.00, 233.82)
	Level 3 (16)	21.69 (16.57, 26.85)	78.89 (46.01, 109.33)
	*c*2	1.893	6.260
	*P*	0.388	0.044

**Table 9:** Expression of ANGPTL4 in serum and urine in patients with proteinuria (HL-Pro/NHL-Pro group) among the different GFR classification groups.

Group (*n*)	GFR (*n*) (ml/min·1.73 m^2^)	Serum ANGPTL4 (ng/ml)	Urine ANGPTL4/Cr (*μ*g/g)
HL-Pro group (*n* = 68)	Phase 1 (44)	21.94 (18.60, 48.85)	50.02 (43.97, 75.11)
	Phase 2 (10)	19.56 (18.26, 25.19)	55.53 (45.25, 107.44)
	Phase 3, 4 (14)	79.70 (21.43, 95.01)	52.39 (49.46, 90.87)
	*c*2	11.842	5.542
	*P*	0.008	0.136

NHL-Pro group (*n* = 46)	Phase 1 (24)	23.70 ± 11.42	33.43 (25.96, 66.33)
	Phase 2 (8)	26.11 ± 8.84	50.43 (44.19, 61.20)
	Phase 3 (10)	20.91 ± 4.84	105.78 (97.34, 112.14)
	Phase 4, 5 (4)	24.27 ± 1.62	64.02 (40.07, 87.97)
	*F*/*c*2	0.362	16.311
	*P*	0.834	0.001

## Data Availability

The data sets used and analyzed in this study are available from the corresponding author on reasonable request.

## Additional Points

*Highlights*. There was no correlation between serum and urine ANGPTL4 levels in patients with hyperlipidemia-related proteinuria. There was a significantly positive correlation between serum ANGPTL4 and blood lipid levels, and urine ANGPTL4 levels exhibit positive correlation with the degree of proteinuria.
